# Survival of two probiotic *Bacillaceae* during processing, storage, and digestion of meat products

**DOI:** 10.1038/s41538-026-00883-8

**Published:** 2026-05-21

**Authors:** Kaan Can, Sümeyye Betül Bozatlı, Abdullah Dikici

**Affiliations:** 1 Institute of Graduate Studies, Usak University, Usak, Turkey; 2 Department of Food Engineering, Faculty of Engineering, Manisa Celal Bayar University, Manisa, Turkey; 3 Department of Food Engineering, Faculty of Engineering and Natural Sciences, Usak University, Usak, Turkey

**Keywords:** Biological techniques, Biotechnology, Microbiology

## Abstract

*Bacillus* and *Clostridium* species have gained attention as probiotic candidates due to their spore-forming ability, which confers high stability during food processing. However, laboratory findings often fail to translate into industrial applications. This study evaluated the feasibility of incorporating *Shouchella clausii* and *Heyndrickxia coagulans* into industrially produced probiotic chicken nuggets and heat-treated chicken sausages. Products were manufactured under standard industrial conditions, cooked, and subsequently reheated according to consumption practices. Probiotic viability was assessed after cooking, during refrigerated storage (+4 °C), and following in vitro digestion. Post-cooking counts remained above 6 log₁₀ CFU/g, with overall survival rates around 85%. Viability was stable throughout storage and was not adversely affected by simulated gastrointestinal conditions. Additionally, probiotic incorporation did not negatively impact color, texture, or sensory properties. These findings demonstrate that widely consumed industrial poultry products can be successfully enriched with probiotic functionality without requiring additional infrastructure or processing modifications.

## Introduction

Spore-forming probiotic bacteria, particularly members of the *Bacillaceae* family, have garnered significant attention in food microbiology due to their remarkable resilience against various processing stresses, including thermal, mechanical, and chemical challenges^1,2^. Unlike conventional probiotic genera such as Lactobacillus and Bifidobacterium, which generally lose viability above 55–60 °C^3^, spore-forming *Bacillaceae* demonstrate superior stability as a consequence of their highly specialized spore structure^1^. Their heat resistance is attributed to a dehydrated core, high levels of dipicolinic acid (DPA), calcium–DPA complexes, a multi-layered cortex and coat, and small acid-soluble spore proteins (SASPs), all of which collectively protect nucleic acids and essential enzymes during exposure to severe processing conditions^1,2^. Consequently, spore-forming probiotics offer a promising alternative for functional food development, especially in non-dairy and processed food matrices where traditional probiotics struggle to maintain viability^4,5^. However, their performance under realistic industrial processing conditions remains insufficiently understood.

Despite the recognized advantages of spore-forming probiotics, their application in complex food systems, particularly meat products, remains poorly characterized under industrial conditions. The food matrix itself, with its specific composition (e.g., fat, protein, water activity, pH), can significantly influence spore survival and subsequent germination dynamics^6,7^. Furthermore, many processed meat products undergo multiple heat treatments an initial cooking during industrial production and a secondary reheating by consumers prior to consumption. While studies have investigated the survival of Bacillus spores under single heat treatments such as pasteurization or baking^8,9^, there remains a critical knowledge gap regarding their viability through such “double cooking” scenarios and subsequent storage in industrially produced meat products.

This study addresses these significant gaps by investigating the potential of two specific spore-forming probiotic strains, *Shouchella clausii* (*S. clausii*, formerly *Bacillus clausii*) and *Heyndrickxia coagulans* (*H. coagulans*, formerly *Bacillus coagulans*), in industrially produced chicken nuggets and heat-treated chicken sausages. These particular strains were selected for their documented probiotic potential and spore-forming capabilities^10,11^. The primary objective was to comprehensively evaluate the survival of these probiotic spores during the entire product lifecycle: initial industrial cooking, extended refrigerated storage, and a subsequent consumer-level reheating process. Additionally, the study assessed the viability of the spores after simulated in vitro gastrointestinal digestion, as well as their impact on the sensory, color, and textural properties of the final products. By focusing on industrially relevant food matrices and a realistic “double cooking” paradigm, this research aims to provide novel insights into the practical application and stability of *S. clausii* and *H. coagulans* in widely consumed meat products, thereby facilitating the development of new functional foods without requiring significant modifications to existing production infrastructure.

## Results

### Viability of probiotic *S. clausii* and *H. coagulans* spores

In this study, the feasibility of using *S. clausii* and *H. coagulans* spores in industrial chicken nuggets and chicken sausage products was investigated. Both food products are packaged and sold after being subjected to heat treatment in the factory, which was named as the “1^st^ cooking” in this study. These products are subjected to another cooking process at consumer’s homes before consumption according to manufacturer’s instructions, which was named as the “2^nd^ cooking” in the study.

During both cooking processes, central temperature was measured with a penetration thermometer (Testo 104-IR, Schwarzwald, Germany). The mean value of central temperature of chicken nuggets was measured as 83.8 ± 1.1 °C during cooking at the factory (1^st^ cooking) and 70.1 ± 0.3 °C after the 2^nd^ cooking process before serving.

Raw chicken nugget dough prepared with or without probiotics (control group) was determined to contain 5.29, 7.26 and 7.20 log₁₀ CFU/g total mesophilic aerobic bacteria (TMAB), *S. clausii* and *H. coagulans*, respectively. The statistically significant change after the inoculation shows probiotic spores were the dominating genus in both products (Table 1).

**Table 1:** Microbiological analysis results of chicken nuggets (log₁₀ CFU/g, mean ± SD)

	Storage (Day)	Control	*S. clausii*	*H. coagulans*
Uncooked	**0**	5.29 ± 0.20^bX^	7.26 ± 0.46^aX^	7.20 ± 0.06^aX^
1^st^ cooking		4.27 ± 0.63^bXY^	6.48 ± 0.06^aY^	6.48 ± 0.21^aY^
2^nd^ cooking		3.86 ± 0.18^bY^	6.21 ± 0.11^aY^	6.16 ± 0.10^aY^
1^st^ cooking	**15**	4.92 ± 0.48^bYZ^	6.44 ± 0.16^aY^	6.47 ± 0.21^aY^
2^nd^ cooking		3.76 ± 0.18^bZ^	6.17 ± 0.11^aY^	6.14 ± 0.10^aY^
1^st^ cooking	**30**	4.50 ± 0.7 ^bZ^	6.52 ± 0.06^aY^	6.48 ± 0.21^aY^
2^nd^ cooking		3.83 ± 0.37^bZ^	6.13 ± 0.21^aY^	6.17 ± 0.10^aY^

^a,b^Values marked with different superscript letters in the same row indicate a statistically significant difference (*p* < 0.05).

^X–Z^Values marked with different superscript letters in the same column indicate a statistically significant difference (*p* < 0.05).

The central temperature of the chicken sausage was measured as 82.5 ± 1.8 °C during cooking at the factory (1st cooking) and 71.5 ± 0.6 °C during the 2nd cooking process before serving. Significant reductions in the spore count were observed after the 1st cooking whereas no significant reduction was determined after the 2nd cooking (Table 2).

**Table 2:** Microbiological analysis results of chicken sausage (log₁₀ CFU/g, mean ± SD)

	Storage (Day)	Control	*S. clausii*	*H. coagulans*
**Uncooked**	**0**	5.49 ± 0.67^bX^	7.31 ± 0.35^aX^	7.19 ± 0.37 ^aX^
1^st^ cooking		4.35 ± 0.63^bXY^	6.51 ± 0.06^aY^	6.49 ± 0.21^aY^
2^nd^ cooking		3.78 ± 0.18^bY^	6.17 ± 0.11^aY^	6.13 ± 0.10^aY^
1^st^ cooking	45	4.18 ± 0.19^bYZ^	6.56 ± 0.18^aY^	6.44 ± 0.08^aY^
2^nd^ cooking		3.68 ± 0.31^bZ^	6.23 ± 0.06^aY^	6.22 ± 0.10^aY^
1^st^ cooking	75	4.09 ± 0.39^bZ^	6.57 ± 0.08^aY^	6.52 ± 0.09^aY^
2^nd^ cooking		3.65 ± 0.31^bZ^	6.23 ± 0.18^aY^	6.08 ± 0.23^aY^

^a,b^Values marked with different superscript letters in the same row indicate a statistically significant difference (*p* < 0.05).

^X–Z^Values marked with different superscript letters in the same column indicate a statistically significant difference (*p* < 0.05).

### Probiotic spore viability during storage

The spore-forming probiotics, which were present at approximately 7 log₁₀ CFU/g in the raw nugget dough, decreased significantly in number to approximately 6.48 log₁₀ CFU/g after the first cooking at the factory (*P* < 0.05). However, no significant decrease in probiotic counts was observed during the second cooking at home (*P* > 0.05) after the chicken nugget was cooled and received by the customer on the same day (Table 1). There were no significant changes in the spore counts after the cooking processes on the 15th and 30th days of chicken nugget storage at +4 °C (Table 1).

Approximately 7.2 log₁₀ CFU/g of probiotics were added to the sausage dough used in heat-treated sausages. After the first heat treatment, the number of spores decreased significantly (*p* < 0.05), falling to approximately 6.5 log₁₀ CFU/g. However, after the second cooking, which was done based on consumer recommendations, no significant decrease was observed, even though the number decreased (Table 2). After storage, the sausages were kept in cold storage for the following days, and analyses on the 45th and 75th days revealed no difference between the first and second cookings (*p* > 0.05) (Table 2).

There was no difference between the 1st and 2nd cooking results of both products on different storage days (*p* > 0.05) (Tables 1 and 2). The thermophilic nature of these strains might have obstructed their germination during storage, thus the number of probiotic organisms remained stable.

### Viability of probiotic spores after in vitro digestion

The results of the in vitro digestion experiments indicate that both probiotic strains retained high viability after exposure to simulated gastrointestinal conditions, with no significant reductions observed (*p* > 0.05, Tables 3, 4).

**Table 3:** Viability of probiotic spores after in vitro digestion of nuggets (log₁₀ CFU/g, mean ± SD)

Time	*S. clausii*	*H. coagulans*
After 2^nd^ cooking	6.10 ± 0.97	6.10 ± 0.18
After Gastric Phase	5.72 ± 0.21	6.02 ± 0.56
After Intestinal Phase	6.15 ± 0.23	6.52 ± 0.38

**Table 4:** Viability of probiotic spores after in vitro digestion of sausage (log₁₀ CFU/g, mean ± SD)

Time	*S. clausii*	*H. coagulans*
After 2nd cooking	6.08 ± 0.36	6.25 ± 0.24
After Gastric Phase	5.93 ± 0.07	5.75 ± 0.24
After Intestinal Phase	6.61 ± 0.26	6.21 ± 0.68

### Effect on sensory, color, and texture properties

The addition of probiotic spores did not result in significant changes in sensory, color, or texture properties of the products (*p* > 0.05, Tables 5–9).

**Table 5:** Effect of probiotic spore addition on sensory properties of chicken nuggets (mean ± SD)

	Control	*S. clausii*	*H. coagulans*
Appearance	7.27 ± 1.44	7.44 ± 1.42	7.25 ± 1.38
Texture	6.75 ± 1.48	6.94 ± 1.56	7.16 ± 1.52
Odor	6.91 ± 1.44	7.13 ± 1.55	7.02 ± 1.48
Taste	6.77 ± 1.28	6.83 ± 1.36	6.80 ± 1.21
General Acceptance	7.05 ± 1.21	7.05 ± 1.35	7.00 ± 1.19

**Table 6:** Effect of probiotic spore addition on sensory properties of chicken sausage (mean ± SD)

	Control	*S. clausii*	*H. coagulans*
Appearance	6.66 ± 1.21	6.96 ± 1.24	6.66 ± 1.12
Texture	6.46 ± 1.40	6.43 ± 1.52	6.20 ± 1.39
Odor	5.93 ± 1.52	6.10 ± 1.66	6.03 ± 1.71
Taste	5.80 ± 1.73	5.83 ± 1.66	6.00 ± 1.64
General Acceptance	6.23 ± 1.38	6.30 ± 1.23	6.40 ± 1.19

**Table 7:** Effect of probiotic spore addition on color parameters (mean ± SD)

Product	Color	Control	*S. clausii*	*H. coagulans*
Nugget (inner surface)	L* (brightness)	63.66 ± 3,48	63.36 ± 3.22	63.67 ± 3.23
	a* (redness)	1.67 ± 0.73	1.63 ± 0.73	1.77 ± 0.66
	b* (yellowness)	14.12 ± 1.37	13.67 ± 1.21	13.79 ± 1.32
Nugget (outer surface)	L* (brightness)	48.60 ± 4.18	49.37 ± 3.77	49.84 ± 3.00
	a* (redness)	5.90 ± 1.27	6.13 ± 1.49	5.84 ± 1.19
	b* (yellowness)	21.84 ± 1.77	22.66 ± 1.21	22.91 ± 1.16
Sausage (outer surface)	L* (brightness)	33.69 ± 1.14	33.09 ± 1.42	32.53 ± 1.49
	a* (redness)	25.57 ± 1.87	24.65 ± 1.74	24.85 ± 2.61
	b* (yellowness)	15.12 ± 1.21	14.83 ± 0.97	14.63 ± 1.20

**Table 8:** Texture profile analysis results of chicken nuggets (mean ± SD)

Parameter	Control	*S. clausii*	*H. coagulans*
Hardness (g)	3040.61 ± 659.47	3028.11 ± 819.90	3059.94 ± 659.47
Chewiness (mJ)	86.41 ± 26.76	86.10 ± 29.21	85.51 ± 27.93
Elasticity (g*s)	0.39 ± 0.01	0.40 ± 0.02	0.39 ± 0.01
Gumminess (g)	2269.68 ± 706.33	2213.35 ± 729.41	2261.40 ± 626.53

**Table 9:** Texture profile analysis results of chicken sausage (mean ± SD)

	Parameter	Control	*S. clausii*	*H. coagulans*
Raw	Hardness (g)	3075.66 ± 1411.86	2785.55 ± 904.25	2825.94 ± 711.26
	Chewiness (mJ)	66.69 ± 26.03	58.05 ± 16.62	64.18 ± 19.97
	Elasticity (g*s)	2.60 ± 0.40	2.41 ± 0.15	2.39 ± 0.19
	Adhesiveness (mJ)	0.74 ± 0.02	0.76 ± 0.04	0.74 ± 0.02
	Extensibility (mm)	0.34 ± 0.03	0.35 ± 0.03	0.35 ± 0.03
Cooked	Hardness (g)	1867.38 ± 967.50	1836.83 ± 862.00	1904.00 ± 1097.78
	Chewiness (mj)	44.89 ± 21.98	42.05 ± 16.35	44.38 ± 20.86
	Elasticity (g*s)	2.37 ± 0.24	2.43 ± 0.21	2.51 ± 0.07
	Adhesiveness (mJ)	0.78 ± 0.26	0.79 ± 0.03	0.77 ± 0.01
	Extensibility (mm)	0.39 ± 0.02	0.42 ± 0.07	0.43 ± 0.12

## Discussion

In both products (Tables 1, 2), probiotic spore count was around the recommended therapeutic level after all cooking processes^12,13^. This study demonstrates that both *S. clausii* and *H. coagulans* maintained high viability throughout industrial processing and subsequent consumer-level heat treatment. Although a statistically significant reduction was observed after the first cooking step, no further significant decrease occurred following the second cooking process. This indicates that the initial thermal exposure represents the primary stress factor, while subsequent reheating exerts a comparatively limited additional impact on spore viability.

From a mechanistic perspective, the observed resilience can be attributed to the intrinsic structural properties of bacterial spores, including their dehydrated core and protective molecular components such as dipicolinic acid (DPA) and spore coat proteins, which collectively enhance resistance to thermal stress^1,2^. The absence of further reduction after the second cooking step may suggest that the surviving spore population represents a more heat-resistant subpopulation, a phenomenon consistent with the heterogeneous resistance reported for *Bacillus* spores^8^.

In addition to intrinsic resistance, the food matrix likely played a critical protective role. Meat products, characterized by high protein and fat content, may provide thermal buffering and reduce heat transfer, thereby enhancing spore survival. Previous studies have shown that formulation parameters significantly influence spore recovery, with higher meat content promoting greater survival^14^. Similarly, matrix composition, including salt concentration and water distribution, has been reported to modulate the thermal resistance of spores^6^.

When compared to the literature, the approximately 85% survival observed in this study is consistent with or higher than previously reported values for similar systems. For example, survival rates of ≥79% for *S. clausii* during baking^9^ and approximately 88% for *H. coagulans* under thermal treatment^15^ have been reported. These findings suggest that the combined effects of industrial processing and food matrix composition in the present study provide conditions that are at least as protective as those reported under controlled experimental settings.

Importantly, probiotic counts remained above the generally recommended therapeutic threshold (>6 log₁₀ CFU/g) after all processing steps, supporting the technological feasibility of incorporating spore-forming probiotics into industrial meat products.

In this study, the stability observed in spores during storage can be explained by the dormant nature of bacterial spores. Under refrigerated conditions, metabolic inactivity likely prevents germination and subsequent cell death, thereby preserving viability. This is consistent with previous reports demonstrating long-term stability of *H. coagulans* spores in various food matrices, including both refrigerated and ambient storage conditions^8,16^.

In addition, the lack of difference between first and second cooking results across storage periods suggests that storage did not induce structural or physiological changes that would increase heat sensitivity. This further supports the robustness of spore-forming probiotics in complex food systems.

The high survival rate of post-digestive spores (Tables 3, 4) can be attributed to their ability to resist acidic stomach conditions and subsequently germinate or remain stable in the intestinal environment. Previous studies have shown that bile salts and changes in pH may trigger spore germination, potentially explaining the slight increases in counts observed after the intestinal phase^7,17^.

Compared to the literature, the survival rates observed in this study are in agreement with or slightly higher than previously reported values. For instance, ≥90% survival of *H. coagulans* has been reported in bread and cheese matrices⁷, while similar stability has been observed for *S. clausii* in bakery products⁹. These findings highlight the suitability of meat products as effective carriers for spore-forming probiotics.

This finding, showing that spores did not affect sensory, color, and textural properties (Tables 5–9) (*p* > 0.05), is consistent with previous studies reporting minimal effects of spore-forming probiotics on food quality characteristics^17–21^.

From a mechanistic standpoint, this can be explained by the metabolically inactive nature of spores under the conditions studied. Unlike vegetative probiotic cells, spores do not actively metabolize substrates or produce compounds that could alter sensory characteristics. As a result, their incorporation into food systems is unlikely to affect product quality, provided that germination is not induced during storage^15,22,23^.

While this study provides valuable insights into the stability of spore-forming probiotics in industrially produced meat products, certain limitations warrant discussion. The absence of detailed physicochemical characterization of the chicken nugget and sausage matrices (e.g., pH, water activity, fat, and protein content) restricts a more precise mechanistic interpretation of the observed spore survival. This limitation should be considered when interpreting the underlying factors contributing to probiotic stability. Additionally, the sensory evaluation was conducted with a relatively small trained panel, which may not fully reflect consumer preferences. Future studies should include larger consumer-based panels to better assess product acceptability.

This study demonstrates that *H. coagulans* and *S. clausii* spores can be successfully incorporated into industrially produced chicken nuggets and sausages. The spores retained >85% viability after double heat treatment. Refrigerated storage at 4 °C did not significantly reduce viability. Simulated gastrointestinal digestion also had no adverse effect. Probiotic incorporation did not alter texture, color, or sensory properties. These products can serve as effective carriers for spore-forming probiotics without requiring major modifications to existing production processes. Although this study confirms technological feasibility, further in vivo studies are needed to evaluate biological activity and health benefits. Overall, spore-forming probiotics offer a robust strategy for developing next-generation functional meat products.

## Methods

### Materials and production of food samples

Materials used in the chicken nugget and sausage recipe were supplied by Gedik Pilic R&D center (Usak, Turkey) and all production steps were carried out at Gedik Pilic (Usak, Turkey) (Tables 10, 11). Commercially available Kerry GanedenBC30® and Enterogermina® were used for *H. coagulans* and *S. clausii*, respectively.

**Table 10:** Chicken nugget product recipe

Material	Proportion (%)
Chicken	82
Flour	12
Black Pepper	0.5
Cumin	0.5
Garlic Powder	1.5
Onion Powder	2.4
Salt	1
Lactic Acid	0.1

**Table 11:** Chicken sausage product recipe

Materials	Proportion (%)
Minced Chicken	75
Beef Fat (kg)	19
Black Pepper	0.5
Cumin	0.9
Pimento	0.25
Salt	2
Red Pepper	0.7
Garlic	1
Sodium Nitrate mg/kg	0.15
Ascorbic acid mg/kg	0.5

The dough of chicken nugget was prepared according to the recipe in Table 10. Then, 7.07 log CFU/g of *S. clausii* spores were added into the dough as a liquid suspension and 7.17 log CFU/g of *H. coagulans* spores were added in lyophilized culture form and the mixing process was carried out.

Patties were shaped in stainless steel molds with a diameter of 4 cm and a thickness of 1 cm. The formed patties were dipped into a liquid coating, consisting of a mixture of flour and water. The coated patties were dipped into a solid-coating consisting of breadcrumbs. Coated patties were fried for 25 s in deep fryer (Frymaster RE14TC, USA) containing sunflower oil at 180 °C and subjected to the cooking process (which is named as 1^st^ cooking) for 8 min in an oven (İnoksan FBE05, Bursa, Turkey) at 95 °C. Chicken nugget samples were packaged under modified atmosphere and stored at + 4 °C for 15 and 30 days.

The dough for heat-treated chicken sausage was prepared according to the recipe at Table 11. 7.3 log CFU/g of *S. clausii* spores were added into the dough as a liquid suspension and 7.17 log CFU/g of *H. coagulans* spores were added in lyophilized culture form and the dough was mixed to achieve homogeneous distribution.

The dough was filled into commercial packaging (Etpak, Elit, Kocaeli, Turkey) containing polyamide and polyethylene suitable for food contact using a manual vertical sausage filling machine (F.Dick, Frankfurt, Germany).

The cased chicken sausage samples were cooked in the oven (İnoksan FBE05, Bursa, Turkey) at 70% humidity and 75% fan speed for 10 min at 55 °C, 10 min at 65 °C and 25 min at 85 °C (1^st^ cooking). After the cooking process, the samples were placed in an ice water bath for rapid cooling and then stored at +4 °C for 45 and 75 days.

Cooked poultry products are usually subjected to another cooking process at consumers’ homes before consumption. Therefore, the chicken nuggets used in our study were cooked in their original form, and the chicken sausages were cut into a standard 15 mm thickness and both samples were cooked according to the manufacturer’s instructions at 180 °C in an oven (Arcelik, AFC 221 HB, Manisa, Turkey) for 6 min (2^nd^ cooking).

### Microbiological analysis

Microbiological enumerations were conducted on days 0, 15, and 30 of storage for chicken nuggets. Chicken sausage samples were taken for enumeration on days 0, 45 and 75 of storage. Microbiological enumerations of both products were conducted on the production day (Day 0) and during storage (15 and 30 days) in order to investigate the effects of 1st and 2nd cooking processes on the viability of probiotic bacteria spores. For microbiological enumerations, 25 g of samples were homogenized with 225 mL of 0.1% sterile peptone water using a stomacher (IUL Instruments, Barcelona, Spain) and decimal dilutions were prepared from this mixture^24^.

### Enumeration of total mesophilic aerobic bacteria

Total Mesophilic Aerobic Bacteria Count was performed in raw samples and cooked control group samples in all groups using Plate Count Agar (Oxoid, Hampshire, United Kingdom) medium. After the plating was performed in duplicate, the plates were incubated at 35 °C for 48 h^25^.

### Enumeration of *H. coagulans* and *S. clausii*

The dilutions were subjected to heat at 80 °C for 10 minutes in a water bath. Then, samples were plated on Mueller-Hinton Agar (Sigma Aldrich, Missouri, USA) and Glucose Yeast Extract BC Agar (HiMedia, USA) for *S. clausii* and *H. coagulans*, respectively. The plates were incubated at 40 °C for 48 h^10^.

### In vitro digestion

For synthetic digestion studies, the in vitro digestion model in the INFOGEST protocol by Brodkorb et al.^26^ was applied with some modifications. Oral fluid (Stimulated Saliva Fluid, SSF), gastric fluid (SGF) and intestinal fluid (Stimulated Intestinal Fluid, SIF) contents were purchased from Sigma Aldrich (USA) and prepared according to Table 12.

**Table 12:** Composition of in vitro digestion fluids

Stimulated Saliva Fluid (SSF)	Stimulated Gastric Fluid (SGF)	Stimulated Intestinal Fluid (SIF)
15.1 mmol/L KCl	6.9 mmol/L KCl	6.8 mmol/L KCl
3.7 mmol/L KH_2_PO_4_	0.9 mmol/L KH_2_PO_4_	0.8 mmol/L KH_2_PO_4_
6.8 mmol/L NaHCO3	12.5 mmol/L NaHCO_3_	42.5 mmol/L NaHCO_3_
0.5 mmol/L MgCl2(H2O)_6_	0.4 mmol/L MgCl_2_(H_2_O)_6_	1.1 mmol/L MgCl_2_(H2O)_6_
0.09 mmol/L HCl	0.13 mmol/L HCl	0.07 mmol/L HCl
0.06 mmol/L (NH4)_2_CO_3_	0.5 mmol/L (NH4)_2_CO_3_	9.6 mmol/L NaCl
0.025 mmol/L CaCl_2_(H_2_O)_2_	11.8 mmol/L NaCl	0.04 mmol/L CaCl_2_(H_2_O)_2_
	0.005 mmol/L CaCl_2_(H_2_O)_2_	10 mM bile salts
	2000U/mL Pepsin	100 U/mL pancreatin

All enzymes, bile salt and CaCl_2_(H_2_O)_2_ was added at the time of the experiment. In the oral phase, a 5-g sample was mixed with 5 mL of SSF and homogenized using a stomacher for 2 min no enzymes were used in the oral digestion, and the final pH value of the mixture was adjusted to 7 with 1 M NaOH. After that in the gastric phase, SGF was added at 1:1 ratio and the pH was adjusted to 3 with 1 M HCl and the mixture was incubated at 37 °C for 2 h. At the intestinal stage, SIF was added to the stomach bolus at 1:1 ratio and the pH was again adjusted to 7 and the mixture was incubated at 37 °C for another 2 h. Samples were taken for plating after the gastric and the intestinal phase.

### Color measurement

For color measurements, Chromometer CR400, Minolta, (Japan) model colorimeter was used. Color parameters L*(brightness), a*(redness-green), b*(yellowness-blue) values were taken into consideration in color measurement. White plate values (L* = 93.3, a*= 0.3162 and b*= 0.3321) were taken as reference for calibration. After cooking the food samples, one measurement was made from 4 different samples from the inner dough and outer coating for chicken nuggets and from the outer surface for chicken sausage. The values were averaged^27^.

### Texture profile analysis

Texture Profile Analysis were performed with Texture Analyzer (Brookfield CT3 4500, MA, USA) and a 4500 g load cell was used. Compression tests were performed on nugget and sausage samples to determine the TPA (texture profile analysis) profiles of the samples. Texture profile analyses were carried out using the software program of the texture analyzer at room temperature (25 °C). Slices of the same thickness (35 ± 1.5 mm in diameter and 15 ± 1 mm) were cut from each sausage sample. All nugget samples were shaped to be in a uniform measurement of 39 ± 1.5 mm in diameter and 11 ± 1 mm in height. 3 samples were taken from each sample and the analyses were carried out. Within the scope of the analysis, 40% compression was applied to nuggets and 20% compression was applied to sausage. Hardness, elasticity, adhesiveness, gumminess and chewiness properties were used as texture parameters^28,29^.

### Sensory analysis

Chicken nuggets and sausage samples were cooked in a 180 °C oven for 6 min before the sensory analysis. 10–12 panelists were selected from trained personnel working at the Usak University Nutrition Services Branch Directorate. The panelists were briefed on the samples and were asked to score each sample on a hedonic scale from 1 to 10 in terms of Appearance, Texture, Odor, Flavor and General Acceptability^30^.

The sensory evaluation was conducted using a trained panel to ensure consistent and reproducible assessment of product attributes. While trained panels are not typically used for consumer acceptance testing, this approach was adopted to obtain controlled and comparative evaluations across samples.

### Statistical analysis

All analyses in production stages were performed with 3 repetitions. ANOVA test was performed on the data of each group by applying the repetition x sample x time model and interactions between variables were calculated. The calculated mean values were separated using Fisher’s least squares method according to General Linear Models (GLM) procedures. The statistical significance level was accepted as 5%. Prior to analysis, the assumptions of ANOVA were assessed, including normality of residuals (Shapiro–Wilk test) and homogeneity of variances (Levene’s test). The data were considered suitable for ANOVA. All statistical calculations were performed using the SAS statistical program and the values were arranged as mean ± standard deviation (mean ± SD)^31^.

## Data Availability

The datasets generated and analyzed during the current study are not publicly available due to restrictions related to ongoing research and institutional policies but are available from the corresponding author on reasonable request.
